# HIV pre-exposure prophylaxis for people who inject drugs: a review of current results and an agenda for future research

**DOI:** 10.7448/IAS.17.1.18899

**Published:** 2014-03-27

**Authors:** Daniel J Escudero, Mark N Lurie, Thomas Kerr, Chanelle J Howe, Brandon DL Marshall

**Affiliations:** 1 Department of Epidemiology, School of Public Health, Brown University Providence, RI, USA; 2 Urban Health Research Initiative, British Columbia Centre for Excellence in HIV/AIDS, Vancouver, BC, Canada; 3 Department of Medicine, University of British Columbia, Vancouver, BC, Canada

**Keywords:** HIV, pre-exposure prophylaxis, injection drug use, people who inject drugs

## Abstract

**Introduction:**

Studies examining the use of pre-exposure prophylaxis (PrEP) to prevent HIV transmission among people who inject drugs (PWIDs) have not been adequately summarized. Recently, the Bangkok Tenofovir Study has shown that PrEP may be effective at reducing new HIV infections among this high-risk group. This randomized controlled trial was the first study to specifically examine the efficacy of PrEP among PWIDs. In this review, we present the current state of evidence regarding the use of PrEP to prevent HIV infection in PWID populations, and set an agenda for future research to inform the most effective implementation of PrEP in the context of existing evidence-based HIV prevention strategies.

**Discussion:**

Despite positive trial results confirming that PrEP may prevent HIV transmission among PWIDs, there remain many questions regarding the interpretation of these results, as well as obstacles to the implementation of PrEP regimens within highly diverse drug-using communities. Aside from the Bangkok Tenofovir Study, we identified only one other published study that has collected empirical data to inform the use of PrEP among PWIDs. The large gap in research regarding the use and implementation of PrEP for PWIDs signals the need for further research and attention.

**Conclusions:**

We recommend that future research efforts focus on elucidating the generalizability of the Bangkok Tenofovir Study results in other injection drug–using populations, examining the willingness of PWIDs to use PrEP in diverse contexts, identifying barriers to adherence to PrEP regimens and determining the most effective ways to implement PrEP programmes within the context of existing evidence-based prevention strategies, including opioid substitution therapy and needle and syringe distribution programmes.

## Introduction

People who inject drugs (PWIDs) continue to be at high risk for HIV-1 infection, with annual parenteral transmission accounting for approximately 7% of new HIV infections in the United States and 10% of new infections globally [1,2]. Although several interventions have been shown to reduce the risk of HIV-1 acquisition among PWIDs and are recommended by the World Health Organization (WHO) and Joint United Nations Programme on HIV/AIDS (UNAIDS) [3], implementation and scale-up remain suboptimal, and consequently many settings continue to experience on-going or accelerating epidemics of HIV among PWIDs [4]. Pre-exposure prophylaxis (PrEP), typically consisting of a once-daily oral tenofovir or tenofovir-emtricitabine regimen, is a potentially promising tool to add to this expanding set of prevention strategies. Several successful PrEP trials have fuelled optimism that PrEP may be effective at reducing transmission among many high-risk groups [5–8], including men who have sex with men (MSM), the negative partner in heterosexual serodiscordant couples and, most recently, PWIDs [8]. However, this optimism has been tempered by disappointing results from multiple unsuccessful trials among women [9,10], as well as a host of implementation issues, including various challenges associated with the scale-up of mass or even targeted administration strategies. The role of PrEP for PWIDs is further complicated by the fact that in many settings, HIV prevention interventions specifically addressing drug users often have little or no public or governmental support [11], and in some places punitive and harsh treatment from government bodies is common and has been linked to poor outcomes [12,13]. Furthermore, concerns regarding HIV prevention trial participation among PWIDs, including trial literacy, persist [14].

The success of PrEP trials among MSM and heterosexual serodiscordant couples (with efficacy results ranging from 39% to 80%) has led the scientific community to begin shifting towards a focus on implementation science regarding the use of PrEP in these populations [15,16]. It is possible that, following positive trial results among PWIDs, a comparable growth in implementation research may occur within communities of PWIDs. Several authors have discussed the potential uses for and barriers to implementation of PrEP among PWIDs [14,17–20], but empirical data collection has only rarely been performed. In anticipation of increased research attention to the implementation of PrEP among PWIDs, as well as continued interpretation of the Bangkok Tenofovir Study results, we present here a review of the current state of evidence for the use of PrEP among PWIDs, and propose an agenda for future research.

## Discussion

A careful review of the published literature revealed only one study outside of the Bangkok Tenofovir Study that collected empirical data on the use of PrEP among PWIDs. This section gives a brief description of these two studies [8,21], followed by a proposed research agenda that would help to address many of the knowledge gaps yet to be studied.

### The Bangkok Tenofovir Study

Of eight articles and abstracts that met our inclusion criteria, seven were analyses of data collected as part of the Bangkok Tenofovir Study (ClinicalTrial.gov identifier NCT00119107) [8,22–27]. Between 2005 and 2010, the Bangkok Tenofovir Study enrolled 2413 PWIDs, who were randomized to either daily oral tenofovir administration or placebo and followed for a maximum of 84 months [8,22,24]. The trial enrolled HIV-negative individuals between 20 and 60 years of age who reported injection drug use during the previous 12 months. To ensure adherence to the study treatments, participants were permitted to choose whether to participate in directly observed therapy (DOT) or retain a study drug diary that would be assessed at monthly visits [25]. A modified intent-to-treat analysis yielded an HIV incidence rate of 0.35 per 100 person-years in the treatment arm, and 0.68 per 100 person-years in the placebo arm, resulting in a PrEP efficacy of 48.9% (95% confidence interval (CI): 9.6%–72.2%) [8]. Those with greater adherence were observed to have a higher level of protection: when restricting analysis to participants with detectable levels of tenofovir, the PrEP efficacy among PWIDs increased from 49% to 74% [8].

### Willingness to use PrEP

The second study identified in our review was an analysis of willingness to use PrEP among PWIDs in Ukraine. Specifically, the authors collected data on the acceptability of PrEP among a sample of Ukrainian PWIDs as part of a multinational survey of the attitudes of high-risk groups (i.e. PWIDs, female sex workers, MSM, heterosexual serodiscordant couples and young women) towards PrEP [21]. Although the study itself consisted of people from these five high-risk groups who were from seven countries, all 128 PWIDs resided in Ukraine.

A majority (53.0%) of the Ukrainian PWIDs responded that they would “definitely” be willing to use PrEP (based on a 4-point Likert scale), and 32.6% responded that they would “probably” be willing to use PrEP. Although these results indicate that most of the Ukrainian PWIDs sampled would be willing to use PrEP, out of all the high-risk groups in the study, they had the highest proportion of participants (6.8%) indicating that they would “definitely not” be willing to use PrEP [21]. In this study, having never injected drugs and currently not injecting drugs were both significantly associated with a higher willingness to use PrEP (both *p*<0.01) [21].

### An agenda for future research and implementation of PrEP among PWIDs

Although several authors have discussed the potential for successful use of PrEP within PWID populations [14,17,18], we have identified a large gap in empirical data that specifically addresses the use and implementation of PrEP among PWIDs.

This dearth of data is concerning given that PWIDs are at high risk of HIV infection in many settings throughout the world [28]. The lack of evidence is also in contrast to the growing bodies of studies conducted in other high-risk groups, including MSM and heterosexual serodiscordant couples, in which willingness to use [29–34], epidemic impact [35–39] and implementation concerns of PrEP have been examined [40–43]. For instance, Campbell *et al*. were able to compile data on attitudes towards and usage of PrEP among 13 different studies of MSM in the United States alone [29].

#### Willingness to use PrEP

Although the efficacy results demonstrated in the Bangkok Tenofovir Study have already prompted the US Centers for Disease Control and Prevention (CDC) to recommend, in interim guidelines, the use of PrEP in very high-risk PWIDs [44], further research is needed on how best to implement PrEP in diverse injecting drug use communities. Specifically, to improve an understanding of the factors associated with willingness to use PrEP among PWIDs, more studies are urgently needed to build upon the Ukrainian data collected by Eisingerich *et al*. [21]. To understand how willingness to use PrEP among PWIDs varies across settings, future research will need to collect data from PWIDs in other regions with high parenteral transmission, such as Asia, Russia and the Middle East. It is possible that context-specific factors, such as epidemic stage, availability of other preventive services (e.g. needle and syringe programmes (NSPs) and opioid substitution therapy (OST)), stigma and discrimination, may affect willingness to use PrEP among PWIDs.

#### Epidemic modelling

Despite several mathematical modelling studies examining the effectiveness and use of PrEP to prevent HIV [35–39], we are unaware of any modelling studies that have estimated the effects of PrEP among PWID populations. Future epidemic modelling studies may be able to provide important insights into the potential implications of specific PrEP strategies to reduce HIV transmission among PWIDs, which could subsequently inform policy and programmes. Furthermore, epidemiological models may be able to estimate many aspects of large-scale PrEP programme implementation for PWID populations, including cost-effectiveness, the effects on incidence and prevalence and the most effective ways to deliver targeted prevention strategies to high-risk persons. With an array of proven HIV prevention methods for PWIDs already available, mathematical models will be essential in determining how PrEP should best be incorporated into larger prevention programmes, and whether adding PrEP to an existing intervention package would be more or less effective than scaling up existing prevention modalities. We thus recommend that mathematical models of PrEP for PWIDs be considered a research priority.

#### Adherence

Additional research should also be conducted on the significance of potentially low adherence among PWIDs who are prescribed PrEP regimens. The Bangkok Tenofovir Study observed that, as with other successful PrEP trials, adherence had a strong, positive influence on PrEP efficacy [8]. When restricting analysis to participants with detectable levels of tenofovir, the PrEP efficacy among PWIDs increased from 49% to 74% [8]. It should be noted, however, that this restriction might have introduced selection bias into the analysis, which could have affected the observed estimate. Overall, the trial investigators were able to achieve a high mean adherence of 83.3% (percentage of days on which participants took oral tenofovir or placebo). However, several measures (likely unavailable in a non-trial setting) were taken by researchers to ensure adequate adherence, including DOT, study drug diaries and contacting participants who had missed appointments [8,25]. Barriers to achieving high adherence and subsequently lower PrEP effectiveness will likely be magnified in real-world settings [15]. The Bangkok Tenofovir Study financially incentivized trial participants to accept and adhere to PrEP; several authors have argued that, outside of a trial setting, barriers to adherence may be a potentially “fatal flaw” of the intervention [18,45]. However, due to a paucity of research, these barriers have yet to be confirmed empirically. In fact, a meta-analysis of adherence to antiretroviral therapy (ART) showed that PWIDs had comparable adherence to non-drug-using populations [46]. These data do not necessarily suggest that PWIDs will have similar PrEP adherence compared to non-drug-using populations, but it does indicate that concerns regarding PWIDs being unable to adhere to PrEP regimens may be unfounded. Future research should examine whether PWIDs will encounter the same barriers to PrEP adherence as they do for ART treatment services, such as interruptions in care due to low social support, criminalization of people who use illicit drugs and incarceration [47]. Although the Bangkok Tenofovir Study was able to negotiate with local corrections officials to maintain PrEP treatment during incarceration [8], this may not be possible in other settings, particularly outside of a trial context. Potentially low adherence among PWIDs not only would decrease PrEP effectiveness but also may increase the risk of developing drug resistance, as was investigated in previous PrEP trials [5–7]. Although the Bangkok Tenofovir Study did not find any tenofovir-associated resistance among those in the trial arm, it should be noted that the incidence of PrEP-associated resistance would need to be high to be detected among only 17 seroconverters in the trial arm (only 15 of whom were tested for resistance) [8]. Subsequently, the risk of PWIDs who are poorly adherent to PrEP developing drug resistance remains an open area of research. To decrease the risk of drug resistance and to maximize effectiveness, initial research into maintaining and improving PrEP adherence among PWIDs should focus on adapting interventions that have been shown to increase ART adherence, including DOT and OST [48,49].

#### PrEP in combination with other prevention methods

Given that PrEP is one modality within a large array of other evidence-based prevention methods, future research should seek to understand when and how best to integrate PrEP within larger prevention programmes. Few HIV interventions are now performed in isolation, and it is unlikely that any one intervention could eliminate the epidemic worldwide among PWIDs [14]. For example, the WHO-UNODC-UNAIDS Technical Guide delineates nine evidence-based interventions to slow the spread of HIV among PWIDs and reduce its associated harms, and it recommends that they be implemented comprehensively and in combination [3]. Given that PrEP has only been shown to at most confer partial protection in all completed clinical trials (among serodiscordant couples, sexually active women, MSM and PWIDs), PrEP will likely be most effective when implemented concomitantly with other prevention modalities. With this in mind, further research is required to investigate how PrEP might best complement existing combinations of evidence-based HIV preventive interventions. Furthermore, the Bangkok Tenofovir Study found PrEP to be efficacious alongside methadone treatment already available at Bangkok Metropolitan Public Health sites [8]; research may need to be conducted on whether other prevention methods, such as NSP (not offered in the trial) [8], impact this observed effect.

Programmes such as NSPs have been shown to reduce population levels of HIV transmission among PWIDs [50], and have benefitted from years of successful implementation and community support. Additionally, the use of sterile injection equipment nearly eliminates the risk of parenteral transmission of HIV (rather than PrEP, which appears to result in a partial reduction, as in the Bangkok Tenofovir Study). However, in some PWID populations, substantial risks of sexual HIV transmission have been observed [51]. The Bangkok Tenofovir Study was not able to distinguish between parenteral and sexual transmission of HIV among study participants; therefore, it is difficult to estimate the benefit of PrEP as a means to prevent sexual transmission in PWID populations. Further research should seek to examine whether PrEP may be used most effectively as a complement to an existing prevention strategy (e.g. high coverage NSPs) to reduce the sexual transmission of HIV among PWIDs.

Research is also required in environments where other prevention strategies either are not being offered to PWIDs or have had limited success with slowing the HIV epidemic. In settings where many evidence-based interventions, including OST and NSPs, are not accessible to PWIDs or are disallowed entirely, PrEP may be an acceptable pharmacological intervention to prevent HIV transmission. Countries with sufficient health infrastructure may be appropriate settings to provide PrEP regimens, such as Russia, which as of 2010 did not offer OST and only provided 7% of its PWID population with access to NSPs annually [11]. Nevertheless, PWIDs frequently lack access to healthcare, and for PrEP to be successful, larger systematic issues such as stigma, discrimination and marginalization experienced by PWIDs who do attempt to access health and social services will need to be addressed. Additionally, given that PrEP confers only partial protection from HIV, significant reductions in incidence in these settings will likely only be achieved with the reform of punitive laws against drug users and the implementation and substantial scale-up of NSPs, OST, HIV treatment and other evidence-based programmes [52]. Accordingly, PrEP alone should not be considered sufficient to prevent or reverse epidemics of HIV infection among PWIDs.

#### Interpreting the Bangkok Tenofovir Study results

Finally, research is needed to understand the generalizability of the Bangkok Tenofovir Study results. A potentially significant limitation of the trial is that risk behaviour decreased substantially as the trial progressed. Only 45% of participants reported injecting drugs at any point during study follow-up [8], and the study relied on participants who had attended Bangkok Metropolitan Public Health sites [8,22], which provide methadone treatment that may have further reduced HIV risk from injection. Additionally, self-reported syringe sharing had decreased to only 2% by the 12th month of follow-up, down from 18% at baseline. This suggests that parenteral transmission may have only contributed a small amount to overall incidence, since risk from injection drug use was low. This study sample may not therefore represent other PWID populations with more consistent injection practices and greater frequency of syringe-sharing behaviour. Furthermore, incident HIV infections in the control arm did not exceed those in the PrEP arm until approximately 3 years into follow-up; such a pattern would suggest non-proportional hazards over the course of the trial and may indicate changing PrEP efficacy over time, which the Cox regression analyses used to determine PrEP efficacy inherently ignore. It should also be noted that this study was not able to determine if particular patterns of injection drug use (i.e. frequency, type of drug) affected intervention efficacy. Lastly, without an appropriate standard of care (e.g. sterile syringe distribution by NSPs) offered to trial participants, it is difficult to understand how PrEP efficacy may also differ in a setting with comprehensive prevention strategies already in place.

## Conclusions

Given that PrEP has been shown to be an efficacious intervention to prevent HIV in high-risk populations, more research is urgently needed to understand the most effective and efficient implementation of PrEP to reduce HIV transmission among PWIDs. We recommend that research focus on elucidating the generalizability of the Bangkok Tenofovir Study results in other PWID populations, examining the willingness of PWIDs to use PrEP in diverse contexts, identifying barriers to adherence to PrEP regimens and determining the most effective ways to implement PrEP programmes within the context of existing evidence-based prevention strategies.
